# Supplementary material for: Electron-hole hybridization in bilayer graphene
Source: Natl Sci Rev. 2019 Dec 19;7(2):248–53. doi: 10.1093/nsr/nwz212 (PMC8288876; doi:10.1093/nsr/nwz212)
Supplement: nwz212_Supplemental_File [file nwz212_supplemental_file.docx]

**Supplementary information for Electron-hole Hybridization in Bilayer Graphene**

Siqi Wang^1, 3^, Mervin Zhao^1^, Changjian Zhang^2^, Sui Yang^1, 3^, Yuan Wang^1, 3^, Kenji Watanabe^4^, Takashi Taniguchi^4^, James Hone^2^, Xiang Zhang^1, 3, 5 *^

**Affiliations**

^1^*NSF Nanoscale Science and Engineering Center (NSEC), 3112 Etcheverry Hall, University of California, Berkeley, California 94720, USA*

^2^*Department of Mechanical Engineering, Columbia University, New York, NY, 10027, USA*

^3^*Materials Sciences Division, Lawrence Berkeley National Laboratory, 1 Cyclotron Road, Berkeley, CA 94720, USA*

^4^*National Institute for Materials Science, 1-1 Namiki, Tsukuba, Japan*

^5^*Faculties of Sciences and Engineering, University of Hong Kong, Hong Kong SAR, China*

*^*^ Correspondence to* [*xzhang@me.berkeley.edu*](mailto:xzhang@me.berkeley.edu)


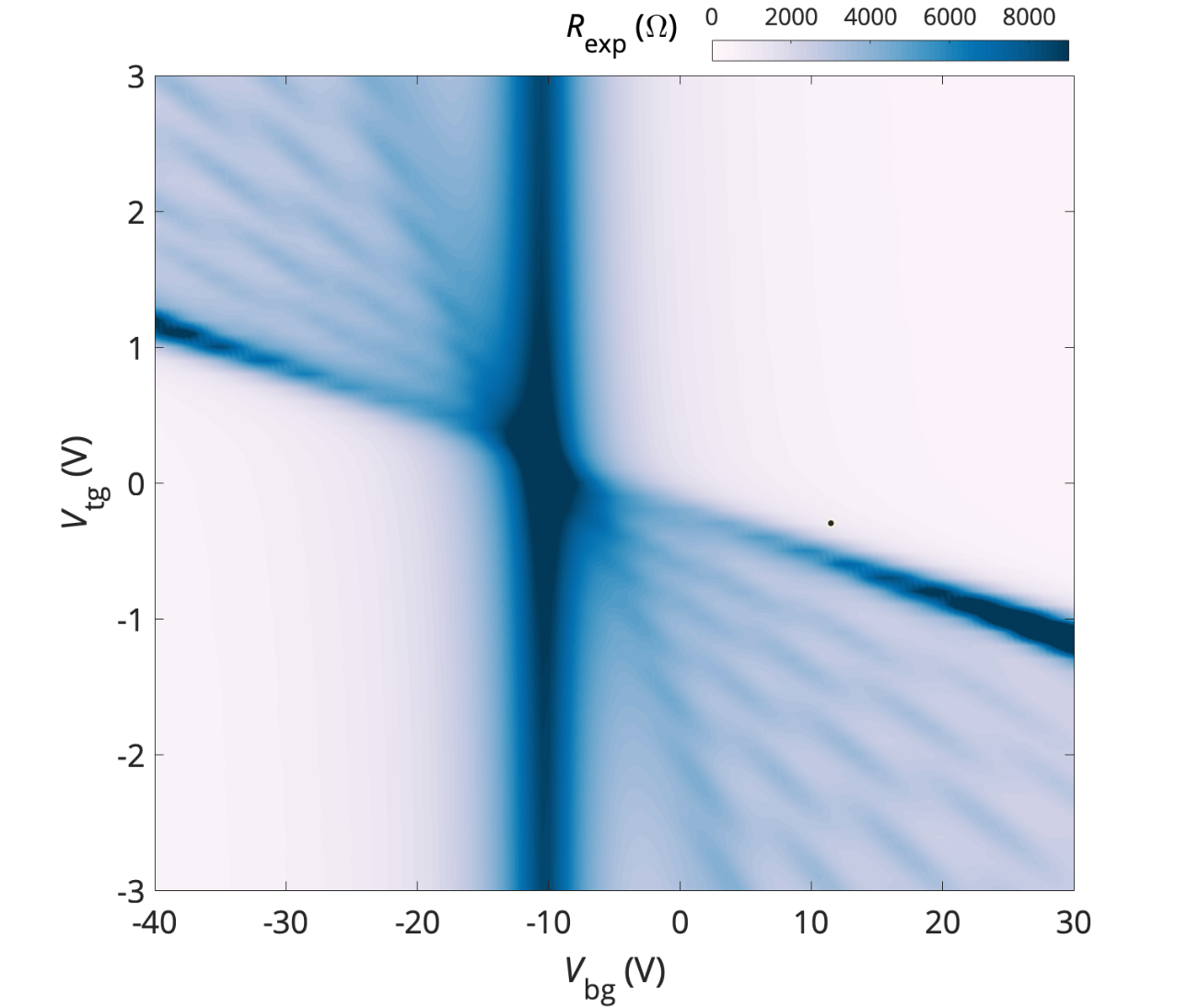


**Figure S1: Resistance mapping on a separate device with Si BG.** Similar resistance modulation as in the main text can be identified.
